# A metal–ion-responsive adhesive material via switching of molecular recognition properties

**DOI:** 10.1038/ncomms5622

**Published:** 2014-08-07

**Authors:** Takashi Nakamura, Yoshinori Takashima, Akihito Hashidzume, Hiroyasu Yamaguchi, Akira Harada

**Affiliations:** 1 Department of Macromolecular Science, Graduate School of Science, Osaka University, 1-1 Machikaneyamacho, Toyonaka, Osaka 560-0043, Japan; 2 Japan Science and Technology Agency (JST), Core Research for Evolutional Science and Technology (CREST), 7 Gobancho, Chiyoda-ku, Tokyo 102-0076, Japan

## Abstract

Common adhesives stick to a wide range of materials immediately after they are applied to the surfaces. To prevent indiscriminate sticking, smart adhesive materials that adhere to a specific target surface only under particular conditions are desired. Here we report a polymer hydrogel modified with both β-cyclodextrin (βCD) and 2,2′-bipyridyl (bpy) moieties (βCD–bpy gel) as a functional adhesive material responding to metal ions as chemical stimuli. The adhesive property of βCD–bpy gel based on interfacial molecular recognition is expressed by complexation of metal ions to bpy that controlled dissociation of supramolecular cross-linking of βCD–bpy. Moreover, adhesion of βCD–bpy gel exhibits selectivity on the kinds of metal ions, depending on the efficiency of metal–bpy complexes in cross-linking. Transduction of two independent chemical signals (metal ions and host–guest interactions) is achieved in this adhesion system, which leads to the development of highly orthogonal macroscopic joining of multiple objects.

## Abstract (web summary)

While common adhesives stick indiscriminately to surfaces, there are many potential applications for smart adhesives that stick to specific surfaces only under certain conditions. Here, the authors report a polymer hydrogel as a functional adhesive material which responds to chemical stimulation by metal ions.

## Graphical abstract

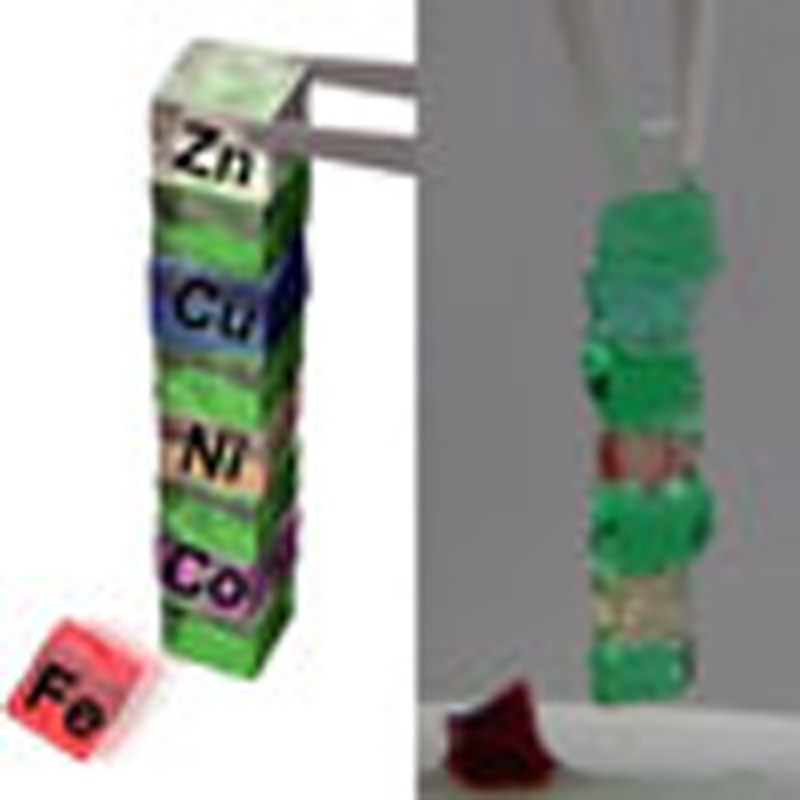

Adhesion of two different materials plays a vital part in a vast field of industries and everyday life^1^,^2^. Reflecting its importance, many theories have been proposed to analyse and explain the mechanism of adhesion, such as mechanical theory, electronic theory, adsorption theory, diffusion theory and chemical bonding theory^3^. Although many attempts have been made to generalize the adhesion process, a consistent explanation is still difficult due to the many factors involved. From a microscopic point of view, some theories attribute the adhesion force to the interatomic forces such as van der Waals interactions and electrostatic interactions. However, discussion based on molecular scales beyond the level of individual functional groups has been hardly brought up. In terms of application, demand is high for smart adhesives that exert their adhesion abilities to a specific surface at a desired timing^4^,^5^,^6^. Adhesives responsive to external stimuli such as pH^7^, light^8^, temperature^9^, humidity^10^ and magnetic force^11^ have been studied and developed, whose switching abilities were achieved mainly by altering the physical nature and morphology of the polymers. To realize precise and orthogonal surface selectivity in adhesion^12^, one promising approach is to utilize chemical interactions at the surface of the adherent. However, partly due to the difficulty in understanding adhesion process, the creation of such intelligent materials with chemical selectivity is still a big challenge.

Recently, we have found adhesion phenomena between soft materials through molecular recognition^13^,^14^,^15^,^16^,^17^. In this case, a polymer hydrogel modified with cyclodextrins as host moieties showed chemically selective adhesion ability to a specific counterpart that possessed complementary hydrophobic guest moieties through host–guest interaction. Furthermore, conversion of the guest moieties in the adhesive targets by external stimuli such as light^18^, pH^19^ and solvent^20^ changed their affinities to cyclodextrin hosts, and realized ON/OFF adhesion switching between the hydrogels. There is also a report on macroscopic coordination bonding of the hydrogels through axial coordination of L-histidine ligand to an iron-porphyrin^21^. In the study, a hydrogel modified with freebase porphyrin did not interact with a gel possessing L-histidine, but addition of FeCl_3_ led to the metalation of porphyrins and the acquisition of its adhesion property.

In the pursuit of smart adhesive materials that stick to a desired target in response to specific stimuli, we have conceived to control molecular recognition ability of hosts in adhesive materials by modifying them with suitable inhibitory guests whose inclusion properties can be switchable. In this work, we focus on metal ions in surrounding environment as chemical stimuli. Metal ions are widely utilized as chemical stimuli to control living organisms^22^, supramolecular systems^23^,^24^,^25^ or properties of soft materials like gels^26^,^27^,^28^,^29^,^30^,^31^,^32^, for their chemical selectivity, versatility and reversible nature of coordination bonds. Herein, we report a metal–ion-responsive functional material that can switch their chemically selective adhesion property by regulating inhibitory inclusion of metal ligands to host moieties. βCD–bpy gel, a polyacrylamide hydrogel modified with both β-cyclodextrin (βCD) moieties and ligands with 2,2′-bipyridyl (bpy) moieties that have a molecular size proper to the cavity of βCD, is created (Fig. 1a). In the hydrogel, the hydrophobic bpy moiety is included in the cavity of βCD to form supramolecular cross-linking^33^,^34^,^35^,^36^,^37^,^38^,^39^,^40^,^41^, βCD–bpy, in the polymer gel, which suppresses the molecular recognition abilities of βCD. On the addition of metal ions, bpy moieties are complexed with them, and thereby charged metal–bpy complexes are released from βCD cavities to form ‘free’ βCD units (Fig. 1b). In the absence of metal ions, βCD–bpy gel (metal–ion-responsive host gel) do not adhere to *t*Bu gel (guest gel), a polyacrylamide gel possessing *t*Bu groups that can be included in βCD. In contrast, in the presence of metal ions reacting with bpy, supramolecular cross-links between βCD and bpy in βCD–bpy gel is dissociated, and adhesion to *t*Bu gel is achieved through the formation of host–guest complex βCD–*t*Bu on the interface of the two gels (Fig. 1c). Furthermore, the adhesive ability of βCD–bpy gel varies with the kinds of metal ions, whose chemical selectivity is explained by the gel’s property change depending on the resulting metal–bpy complexes.

## Results

### Preparation of βCD–bpy gel

Polyacrylamide was selected as the main chain in this study because interactions of –CONH_2_ groups with cyclodextrins^13^ or metal ions^42^ are known to be small. βCD–bpy gel was prepared by radical copolymerization of acrylamide (AAm), mono(6-deoxyacrylamido)-β-cyclodextrin (βCDAAm)^13^, 5-acrylamidomethyl-5′-methyl-2,2′-bipyridine (bpyAAm) and *N*,*N*′-methylenebisacrylamide (MBAAm) in dimethyl sulfoxide (DMSO), followed by the replacement with H_2_O and used as a hydrogel. The feed ratio of the monomers used in the preparation was optimized as follows to realize suitable supramolecular cross-linking and hardness of the gel (Supplementary Table 1): AAm: 92 mol%, βCDAAm: 3 mol%, bpyAAm: 3 mol%, MBAAm 2 mol%. βCD–bpy gel was characterized by ^1^H solid-state field-gradient magic angle-spinning (FG-MAS) NMR measurements (Fig. 2a). The signals for both βCD and bpy moieties were observed, which confirmed the introduction of both groups into the acrylamide polymer gel scaffold. From integral ratios of the signals, the mol% contents were determined to be 2.2 and 3.0 mol% for βCD and bpy moieties, respectively. These values were also supported by elemental analysis (Methods). As reference materials, βCD gel, bpy gel and AAm gel were also prepared according to the same procedure with βCD–bpy gel (Fig. 2b).

### Supramolecular cross-linking in βCD–bpy gel

The inclusion properties of βCD and bpy were investigated on small molecules in solution. The binding constant *K*_a_ [M^−1^] between 2,2′-bipyridyl and βCD was determined to be 1.0 × 10^2^ (D_2_O, 298 K) by a ^1^H NMR titration experiment (Supplementary Figs 1,2)^43^, which showed a modest affinity of 2,2′-bipyridyl to the cavity of βCD. The inclusion was also confirmed by using their acrylamide derivatives, βCDAAm and bpyAAm (Supplementary Fig. 3). Although bpyAAm was almost insoluble in D_2_O at room temperature, addition of 1 eq. of βCDAAm (10 mM) dissolved 10% of bpyAAm through the formation of inclusion complexes βCDAAm–bpyAAm. These data show that bpy can be included in the βCD’s cavity, and that it acts as an inhibitor to βCD as a host.

Supramolecular cross-linking of βCD–bpy in βCD–bpy gel was investigated. Figure 2c showed the swelling ratios *Q* (= (the weight of a swollen gel)/(the weight of the corresponding dried gel) of βCD–bpy gel and its reference gels in DMSO and H_2_O, respectively. After replacing the solvent from DMSO to H_2_O, βCD–bpy gel was largely contracted (*Q*=25.1 and=6.7 for DMSO and H_2_O, respectively) compared with the other gels. This contraction is explained by an increase in crosslink density on the formation of the inclusion complex βCD–bpy in the polymer network. To demonstrate supramolecular cross-linking in βCD–bpy gel, the size change of the gel on treatment with organic molecules that disrupt the formation of the host–guest complex was investigated (Fig. 2d,e). After immersing in a 10-mM aqueous solution of βCD (a competitive host) or 1-admantanecarboxylic acid sodium salt (AdCANa, a competitive guest), βCD–bpy gel was swollen up to 110±2 or 161±5% (length ratio), respectively. The expansion of βCD–bpy gel was larger than that of the other gels, except in the case of bpy gel immersed in βCD solution, which can be explained by change in polymer’s hydrophilicity caused from βCD’s encapsulation of hydrophobic bpy side chain. These observations indicate that the supramolecular cross-linking βCD–bpy that had existed in βCD–bpy gel was dissociated by appropriate external stimuli, which caused the gel to swell.

### Property changes of βCD–bpy gels depending on metal ions

**Figure 1:**
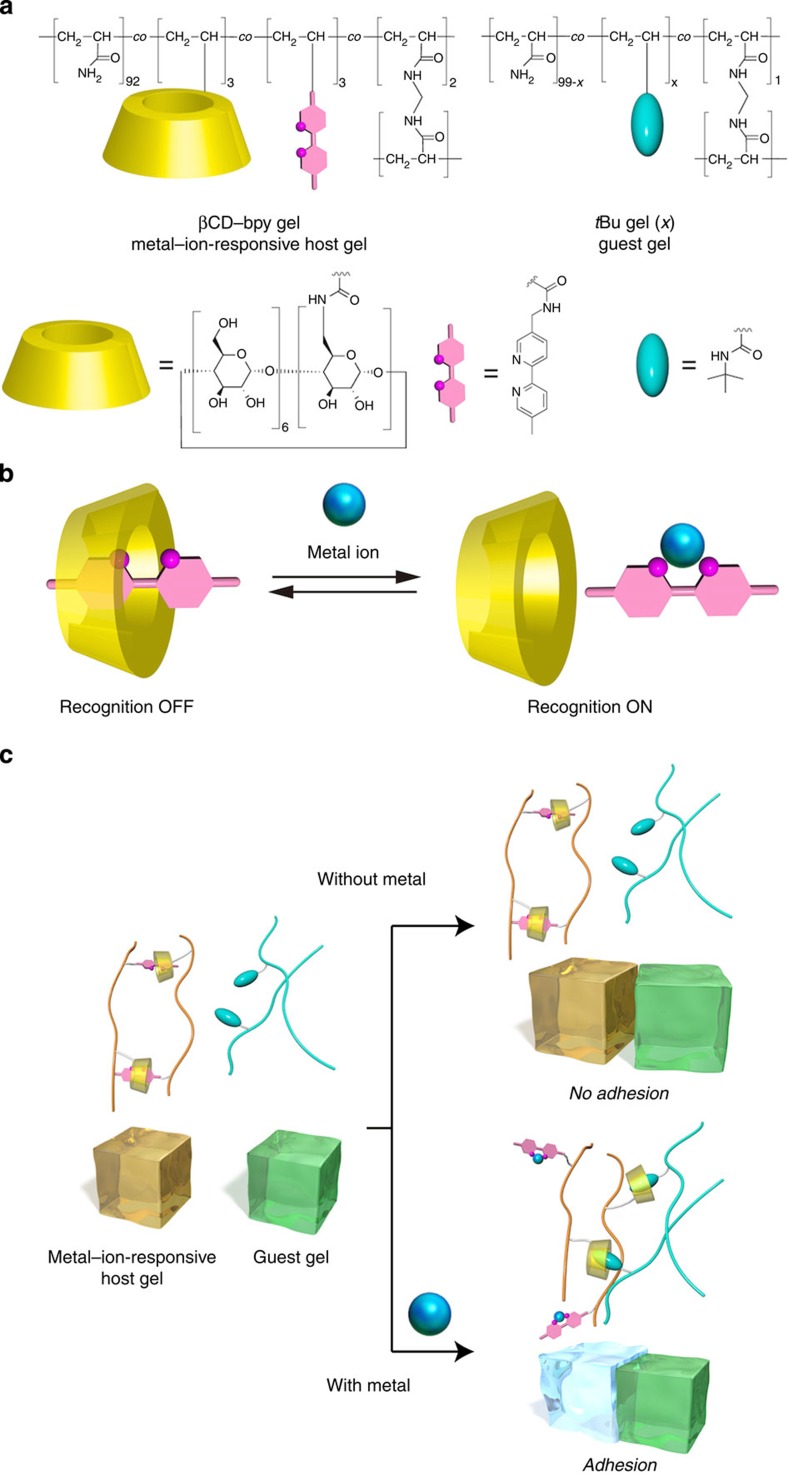
A metal–ion-responsive adhesive material. (**a**) Chemical structures of a metal–ion-responsive host gel (βCD–bpy gel) and a guest gel (*t*Bu gel (*x*)). Here, *x* represents the mol% content of *N*-*t*BuAAm groups in the guest gel. (**b**) Schematic representation of the switching of molecular recognition property of βCD via inhibitory inclusion of bpy and its release by complexation to a metal ion. (**c**) Adhesion of the metal–ion-responsive host gel (βCD–bpy gel) to the guest gel (*t*Bu gel (*x*)) induced by metal ions as chemical stimuli.

As efficient formation of βCD–bpy units in βCD–bpy gel was demonstrated, the reaction of metal ions with the βCD–bpy inclusion complex was investigated. As a model experiment to obtain detailed information about the reaction, the effect of Zn^2+^ ion on a βCD–bpy inclusion complex was examined by ^1^H NMR measurements in D_2_O solution (Supplementary Fig. 4). On the addition of Zn^2+^, bpy reacted with Zn^2+^ and was released from βCD, which made the cavity of βCD free. Subsequent removal of Zn^2+^ from bpy with a strong metal chelator, ethylenediaminetetraacetic acid tetrasodium salt (EDTA·4Na), regenerated the inclusion complex βCD–bpy. It was demonstrated that inclusion and release of bpy with βCD were reversibly controlled by the presence of the metal ion (Fig. 1b).

The reaction of metal ions with βCD–bpy gel was investigated by immersing the gel in corresponding metal chloride salt aqueous solutions (Fig. 3). The gel changed its properties reflecting chemical characteristics of different metal–bpy complexes. Figure 3a shows appearance change of βCD–bpy gel observed by microscopy after immersing the gel in CuCl_2_ or FeCl_2_ aqueous solution. After treatment with Cu^2+^, the gel became highly swollen up to *ca.* 300% in length. In the case of Fe^2+^, the gel was modestly expanded (*ca.* 120%) and its colour turned from colourless to red. The red colour is attributed to tris(bpy) complex [Fe(bpy)_3_]^2+^ with metal-to-ligand charge transfer absorption. Figure 3b shows the length change of βCD–bpy gel after treatment with various metal chloride salt solutions. About three equivalents of metal ions were added against the bpy moieties in the gel. The swelling behaviour was very different depending on the type of metal ions. The largest expansion was observed when reacted with Cu^2+^ (310±20%), while treatment with Fe^2+^ did not largely expand the gel (120±1%), and the gel was almost the same in length in the case of Mg^2+^ (96.3±0.3%). Hardness of the gel was also changed on reaction with metal ions. Figure 3c shows data for tensile strength measurements of βCD–bpy gel before and after treatment with metal ions. The tensile modulus of the βCD–bpy gel was 7.2±0.4 kPa, and it was decreased after the reaction with Cu^2+^ (3.0±0.2 kPa). Meanwhile, the tensile modulus was highly increased after the reaction with Fe^2+^ (88±1 kPa).

This selectivity is well explained by the difference in metal-to-ligand ratio of the resulting metal–bpy complexes and their efficiency in cross-linking (Fig. 3d; See also Supplementary Fig. 5 and Supplementary Table 2 for the equilibrium between metal–bpy complexes of each element). First, the complexation constant of Mg^2+^ and 2,2′-bipyridyl is negligible, and that of Mn^2+^ and bpy is relatively small (log *β*_3_=5.9; ref. 44). These metal ions only slightly interact with the polymer framework of βCD–bpy gel and thus its size change was small. Second, metal ions such as Co^2+^, Ni^2+^, Cu^2+^ and Zn^2+^ mainly form mono(bpy) complex [M(bpy)X_*x*_]^*n*+^ (X=Cl^−^ or H_2_O) when the amount of metal ions is larger than that of bpy (Supplementary Fig. 5)^44^. Thus, the metal ions dissociated the inclusion complex βCD–bpy into mono(bpy) metal complex and free βCD, which decreased in the number of cross-link points. This decross-linking together with an increased swelling pressure brought about by positive charges of metal–bpy complexes made βCD–bpy gel to largely expand and become soft. And thirdly, Fe^2+^ ion mainly forms the tris(bpy) complex [Fe(bpy)_3_]^2+^ even when the excess amount of Fe^2+^ ions against bpy is present in the system (Supplementary Fig. 5)^44^. Thus, the reaction of Fe^2+^ to βCD–bpy gel replaced the cross-links of the inclusion complex βCD–bpy with those of the tris(bpy) complex [Fe(bpy)_3_]^2+^. This tris(bpy) complex working as three-way junction in the gel network^45^ counteracted the increasing swelling pressure caused by positive charges and contributed to the stiffness of the gel. To summarize, βCD–bpy gel behaved as an environmentally responsive material that altered its property based on the characteristics of the metal ions as chemical stimuli.

### Metal–ion-responsive adhesion through molecular recognition

We investigated the adhesion property of βCD–bpy gel and its metal ion responsiveness. Here, *t*Bu gels was selected as a subject material to adhere as a model for chemically selective adhesion (Fig. 1a)^13^. It was expected that βCD–bpy gel (‘metal–ion-responsive host gel’) adhered to *t*Bu gel (‘guest gel’) via the formation of inclusion complexes βCD–*t*Bu on the interface of the gels. *t*Bu gels (*x*) were prepared by radical copolymerization of AAm, *N*-*tert*-butylacrylamide (*t*BuAAm) and MBAAm in DMSO and used as hydrogels by replacing the solvent with water. Here, *x* represents the mol% content of *N*-*t*BuAAm in feed on the preparation of the gel. Inclusion of *t*Bu group within the cavity of βCD was tested in a model experiment in aqueous solution, and its binding strength was comparable to that of bpy groups (binding constant *K*_a_ of *t*BuAAm to βCD: 1.0 × 10^2^ M^–1^ (D_2_O, 298 K), (Supplementary Figs 6,7)).

The adhesion between βCD–bpy gel and *t*Bu gel (20), and the effect of metal ions on it were studied (Fig. 4a–c). βCD–bpy gel did not adhere to *t*Bu gel (20) in the absence of metal ions (Fig. 4b). This is because inclusion ability of βCD moieties was suppressed by efficient formation of βCD–bpy cross-linking in the gel. However, after immersing a piece of βCD–bpy gel in 100 mM CuCl_2_ aqueous solution for 1 min, it acquired the adhesion ability to that of *t*Bu gel (Fig. 4a; Supplementary Movie 1). The adhesion was so strong that the piece of *t*Bu gel was lifted up. It was suggested that Cu^2+^ ions dissociated βCD–bpy units and generated free βCD that could engage in the formation of inclusion complex with *t*Bu groups, and adhesion between the two gels was achieved via the interfacial linking by βCD–*t*Bu units (Fig. 1c). Furthermore, the removal of Cu^2+^ from βCD–bpy gel by immersing in a 100 mM aqueous solution of EDTA·4Na as a metal ion chelator lost its adhesion ability, which means that reversible switching of adhesion properties in response to metal ions was achieved via control of dissociation and reformation of βCD–bpy units in the gel.

### Adhesion strength of βCD–bpy gel

The adhesion property of βCD–bpy gel showed metal ion selectivity. βCD–bpy gel immersed in 100 mM FeCl_2_ aqueous solution did not adhere to *t*Bu gel (Fig. 4c), which was in clear contrast to the case of CuCl_2_. Figure 4d shows results of tensile adhesion strength measurements between βCD–bpy gels and *t*Bu gels (10) immersed in various kinds of metal chloride salt solutions ([MCl_2_]=10 mM (M^2+^=metal ion, 3 eq [/bpy]). The pieces of both gels were immersed for 3 h to reach their equilibrium. See the Methods section for details). βCD–bpy gel treated with Co^2+^, Ni^2+^, Cu^2+^ and Zn^2+^ exhibited adhesion ability to *t*Bu gel (10), with the highest value (1020±20 Pa) of Zn^2+^. In contrast, the gels treated with Mg^2+^, Mn^2+^ and Fe^2+^ did not adhere to it. This selectivity on the kind of metal ions can be explained by the change in gel’s physical property as discussed above (Fig. 3d). (1) Metal ions such as Mg^2+^ and Mn^2+^ that do not interact at all or only slightly interact with bpy groups had little or no effect on the cross-linking of βCD–bpy units of βCD–bpy gel, hence adhesion ability based on βCD’s molecular recognition was kept suppressed. (2) Metal ions such as Co^2+^, Ni^2+^, Cu^2+^ and Zn^2+^ that form mono(bpy) complex [M(bpy)X_*x*_]^*n*+^ (X=Cl^−^ or H_2_O) generated free βCD in the gel, which gave its adhesion property based on molecular recognition. In addition, decross-linking of network made the gel soft and raised the mobility of the βCD moiety on the polymer scaffold, which contributed the efficient formation of βCD–*t*Bu inclusion complexes on the interface. (3) Fe^2+^ ion, which mainly forms the tris(bpy) complex [Fe(bpy)_3_]^2+^, dissociated the inclusion complex βCD–bpy into the tris(bpy) iron complex and the βCD with its cavity free. Although free βCD moieties were generated, the three-way cross-link by tris(bpy) complex made the gel harder over 10-fold (Fig. 3c) and decreased the mobility of βCD units, thus its adhesion ability was impaired.

The mechanism of the metal–ion-responsive adhesion based on switching of molecular recognition was further supported by quantitative adhesion measurements. Figure 4e shows the results of tensile adhesion measurements between βCD–bpy gel/*t*Bu gel (10) immersed in CuCl_2_ aqueous solutions with different concentrations. βCD–bpy gel did not exhibit its adhesion property without Cu^2+^ or with small amount of Cu^2+^. Meanwhile, the gel immersed in 0.1 mM solution of CuCl_2_ (about 0.2 eq [/bpy]) showed weak adhesion (220±30 Pa) to *t*Bu gel, which suggested that the inclusion complexes βCD–bpy near the gel’s surface were dissociated. The adhesion became stronger as the solution of CuCl_2_ was more concentrated, and the measured adhesion strength was as high as 1,000±200 Pa when immersed in a 100-mM solution of CuCl_2_. Furthermore, treatment of βCD–bpy gel preimmersed in a 10-mM solution of CuCl_2_ with a 100-mM solution of EDTA·4Na lost its adhesion ability to *t*Bu gel. This data can be explained by the increase in the number of free βCD as the metal complexation reaction with bpy proceeded. Interfacial formation of βCD–*t*Bu between two gels was supported by quantitative tensile adhesion measurements using *t*Bu gels (*x*) with different mol% contents of *t*Bu groups (Fig. 4f)^15^. βCD–bpy gel immersed in CuCl_2_ solution did not adhere to the AAm gel without *t*Bu groups, so there was no macroscopic attractive interaction between polyacrylamide scaffolds of the two gels. The figure shows that *t*Bu gel (*x*) possessing more *t*Bu groups gave a higher adhesion strength with βCD–bpy gel (1200±100 Pa for *x*=25), which suggested that adhesion strength is dependent on the number of inclusion complexes formed between the gels. The formation of inclusion complex βCD–*t*Bu on the interface was further supported by the experiments using *t*BuOH as competing guests for βCD (binding constant *K*_a_ of *t*BuOH to βCD: 4.8 × 10 M^–1^ (H_2_O, 298 K)^46^). The pieces of the two gels did not adhere to each other in the presence of *t*BuOH as an inhibitor in the immersing solution (1,000 mM). These experiments demonstrated the importance of βCD’s molecular recognition in the adhesion process.

## Discussion

We have reported βCD–bpy gel as a functional soft material that exerts its adhesion ability via switching of molecular recognition property. The regulation of adhesion based on the βCD’s inclusion ability was realized by the effective formation and dissociation of supramolecular cross-linking βCD–bpy in response to metal ions as chemical stimuli. Furthermore, βCD–bpy gel exhibited selectivity on the kind of metal ion in its adhesion, which was derived from the difference in cross-linking efficiency of metal–bpy complexes.

In this system, initially suppressed adhesion property of the material was triggered by external stimuli, and the acquirement of ability was unaccompanied by drastic phase change of the adhesive substances (from liquid to solid, and so on). This makes a clear departure from the mechanism of normal adhesive materials like glues. It is worth noting that chemically selective adhesion by molecular recognition was expressed by the kind of metal ion, which is also a chemical signal. In other words, transduction of two independent chemical signals was achieved in this adhesion system. Using the principle, highly orthogonal macroscopic joining of multiple objects can be created.

The metal–ion-responsive adhesive material can be utilized for many applications. For example, utilizing its reversible adhesion, one could incorporate it into a biomimetic soft robot^47^ that can grab and detach a target object in response to metal ions that are fed either internally (resembling blood circulation) or externally (from environment). Another example would be a smart reinforcement/masking material that detects metal ions leaked from containers or pipes, and sticks to the weaken part specifically. Furthermore, in combination with other stimuli response^13^,^14^,^15^,^16^,^17^,^18^,^19^,^20^,^21^, medical applications such as nanogels for drug delivery systems^48^,^49^ would be possible by making use of its selectivity and adhesion strength in wet conditions. We hope that the concept presented here, that is, chemically selective adhesives with chemical stimuli responsiveness, inspire creativity in various fields of science and engineering.

## Methods

### General

Unless otherwise noted, solvents and reagents were purchased from Nacalai Tesque Inc., Tokyo Chemical Industries Co. Ltd., Wako Pure Chemical Industries Ltd., Sigma-Aldrich Co. or Merck Ltd. and used without further purification. Water used for the preparation of the aqueous solutions was purified with a Millipore Elix 5 system.

5-Aminomethyl-5′-methyl-2,2′-bipyridine used for the synthesis of 5-acrylamidomethyl-5′-methyl-2,2′-bipyridine was synthesized from 5,5′-dimethyl-2,2′-bipyridine in three steps according to the literature^50^. Mono(6-deoxyacrylamido)-β-cyclodextrin (βCDAAm) was synthesized according to the literature^13^. Elemental analysis (calcd., found for C_45_H_78_N_1_O_37.5_ ((βCDAAm)·(H_2_O)_2.5_): C (43.83, 43.73), H (6.38, 6.23), N (1.14, 1.20). β-Cyclodextrin was purchased from Junsei Chemical Co. Ltd., and recrystallized from water. Elemental analysis: (calcd., found) for C_42_H_84_O_42_ ((βCD)·(H_2_O)_7_): C (40.00, 39.81), H (6.71, 6.57). AdCANa was prepared by neutralization of 1-adamantanecarboxylic acid (Tokyo Chemical Industries Co. Ltd.) with equivalent amount of NaOH.

The ^1^H NMR and ^13^C NMR spectra were recorded on a JEOL ECA-500 or a JEOL ECS-400 NMR spectrometers. Residual solvent signals were used for calibration of ^1^H NMR (DMSO-*d*_6_ (δ 2.50 p.p.m.) and D_2_O (4.77 p.p.m.)) and ^13^C NMR (DMSO-*d*_6_ (δ 39.52 p.p.m.))^51^. The solid-state ^1^H FG-MAS NMR spectra were recorded on a JEOL ECA-400 NMR spectrometer. Sample spinning rate was 7 kHz. Electrospray ionization-time-of-flight mass spectrometry spectrum was measured using Orbital XL (Thermo Fisher Scientific). Sizes of the gels were measured using an EVOS AME i2111 digital inverted microscope (Life Technologies). Tensile adhesion strength and mechanical properties of gels were measured by a Yamaden RE2–33005C creep metre.

### Synthesis of bpyAAm

To a Schlenk flask filled with argon gas were added 5-aminomethyl-5′-methyl-2,2′-bipyridine (100.5 mg, 0.504 mmol, 1.0 eq), dehydrated tetrahydrofuran (4 ml) and triethylamine (Et_3_N; 0.14 ml, 1.00 mmol, 2.0 eq). The mixture was stirred at 0 °C and acryloyl chloride (61 μl, 0.75 mmol, 1.5 eq) was added dropwise. The reaction mixture was warmed to room temperature and stirred for 2 h. The mixture was filtrated, and the filtrate was concentrated to dryness under reduced pressure. The crude product was purified using flash column chromatography (silica gel, eluent: CHCl_3_/CH_3_OH). 5-Acrylamidomethyl-5′-methyl-2,2′-bipyridine was obtained as a colourless solid (85.4 mg, 67%). See Supplementary Figs 8,9 for ^1^H NMR and ^13^C NMR spectra, respectively. M.p.: 167.5–168.0 °C; ^1^H NMR (CDCl_3_, 500 MHz, 298 K): *δ* 8.55 (1H, d, *J*=2.3 Hz), 8.49 (1H, d, *J*=2.3 Hz), 8.28 (1H, d, *J*=8.0 Hz), 8.23 (1H, d, *J*=8.0 Hz), 7.72 (1H, dd, *J*=8.0, 2.3 Hz), 7.61 (1H, ddd, *J*=8.0, 2.3, 0.6 Hz), 6.35 (1H, dd, *J*=16.9, 1.4 Hz), 6.26 (1H, br), 6.15 (1H, dd, *J*=16.9, 10.3 Hz), 5.69 (1H, dd, *J*=10.3, 1.4 Hz), 4.56 (2H, d, *J*=5.7 Hz), 2.39 (3H, s); ^13^C NMR (CDCl_3_, 126 MHz, 298 K): *δ* 165.6, 155.7, 153.3, 149.6, 148.6, 137.5, 136.6, 133.6, 133.5, 130.4, 127.3, 120.7, 120.7, 40.9, 18.4; HRMS (*m*/*z*): [M+H]^+^ calcd. for C_15_H_16_N_3_O, 254.1288; found, 254.1295; Elemental analysis (calcd., found for C_15_H_15.2_N_3_O_1.1_ ((bpyAAm)·(H_2_O)_0.1_)): C (70.62, 70.49), H (6.01, 5.94), N (16.45, 16.21).

### Preparation of βCD–bpy gel

**Figure 2:**
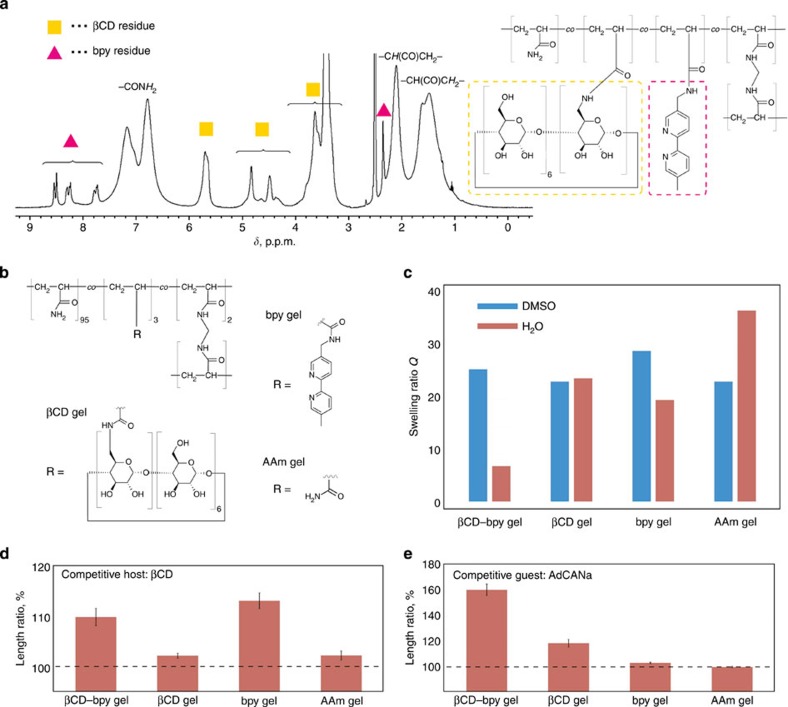
Characterization of βCD–bpy gel and supramolecular cross-linking in the gel. (**a**) FG-MAS NMR of βCD–bpy gel swollen with DMSO-*d*_6_. (**b**–**e**) Supramolecular cross-linking in βCD–bpy gel. (**b**) Chemical structures of βCD gel, bpy gel and AAm gel as reference gels for βCD–bpy gel. (**c**) Swelling ratio *Q* of gels immersed in DMSO and H_2_O, respectively. Here, *Q*=(the weight of a swollen gel)/(the weight of the corresponding dried gel). (**d**,**e**) Length changes of gels by immersing in aqueous solutions of competitive host (βCD) and guest (AdCANa) molecules (10 mM) to disrupt supramolecular cross-linking (error bars, s.e.m. (*n*=4)).

**Figure 3:**
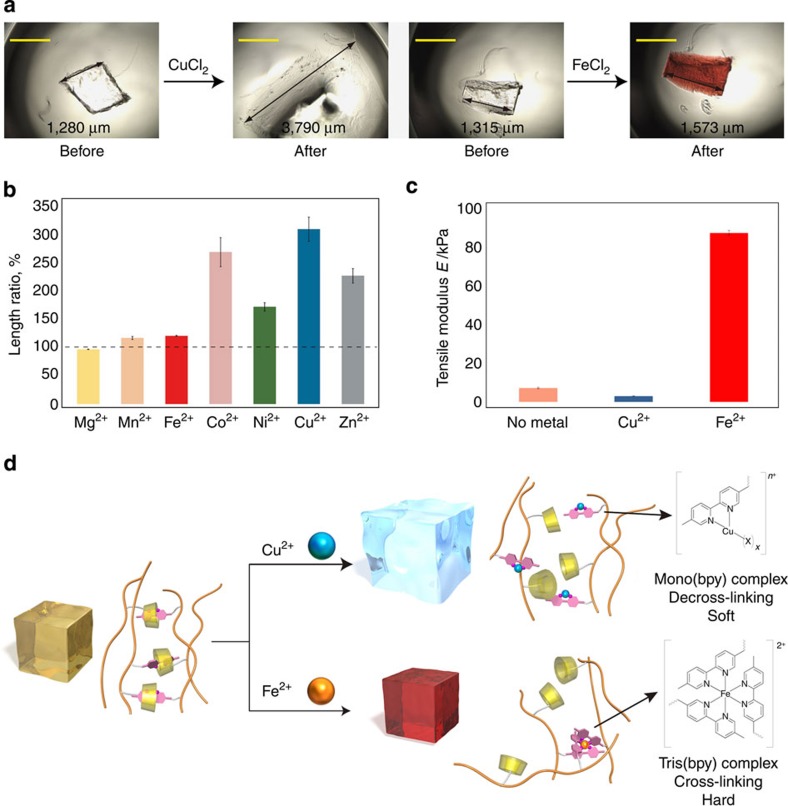
Reaction of metal ions with βCD–bpy gel and its property change. (**a**) Photographs of pieces of βCD–bpy gel before and after immersion in an aqueous solution of CuCl_2_ or FeCl_2_. Scale bar, 1 mm. (**b**) Length change of βCD–bpy gel on reaction with various metal salt aqueous solution ([MCl_2_]=2 mM (M^2+^=metal ion, 3 eq [/bpy]). Error bars, s.e.m. (*n*=4)). (**c**) Tensile modulus of βCD–bpy gels before and after immersed in an aqueous solution of CuCl_2_ or FeCl_2_ ([MCl_2_]=10 mM (M^2+^=metal ion, 3 eq [/bpy]). Error bars, s.e.m. (*n*=4)). (**d**) Schematic representation of decross-linking or cross-linking of βCD–bpy gels by metal–bpy complex formation.

A representative procedure: to a sample tube (φ 19 mm) was added 1,000 μl of DMSO solution containing AAm (1,840 μmol), βCDAAm (60 μmol), bpyAAm (60 μmol), MBAAm (40 μmol; total monomer concentration: 2.00 M) and AIBN (20 μmol). The solution was deaerated by bubbling argon gas through it for 30 min. The mixture was heated in 65 °C oven for 24 h to form a gel. The gel was washed with DMSO (soaked and shaken in DMSO solvent) for 12 h and this was repeated four times, to give a gel swollen with DMSO (4,046 mg). After that the gel was washed with H_2_O (4 days, H_2_O was replaced once a day) and used as a hydrogel (1,081 mg). The swelling ratio in Fig. 2b was calculated based on the weight of hydrogels after freeze dried. See Fig. 2a for FG-MAS ^1^H NMR spectrum of the gel immersed in DMSO-*d*_6_. Elemental analysis of the hydrogel after freeze dried: (calcd., found) for C_436.4_H_779.6_N_108_O_221.8_ ((AAm)_92.8_(βCDAAm)_2.2_(bpyAAm)_3.0_(MBAAm)_2.0_(H_2_O)_45_): C (47.27, 47.28), H (7.05, 6.98), N (13.64, 13.87).

### Preparation of βCD gel

βCD gel was prepared according to a similar procedure for βCD–bpy gel, using 1,000 μl DMSO solution of AAm (1,900 μmol), βCDAAm (60 μmol), MBAAm (40 μmol) (total monomer concentration: 2.00 M) and AIBN (20 μmol). The weights of a βCD gel swollen in DMSO and H_2_O were 3,527 and 3,632 mg, respectively.

### Preparation of bpy gel

Bpy gel was prepared according to a similar procedure for βCD–bpy gel, using 1,000 μl DMSO solution of AAm (1,900 μmol), bpyAAm (60 μmol), MBAAm (40 μmol) (total monomer concentration: 2.00 M) and AIBN (20 μmol). The weights of a bpy gel swollen in DMSO and H_2_O were 3,133 and 2,114 mg, respectively.

### Preparation of AAm gel

AAm gel was prepared according to a similar procedure for βCD–bpy gel, using 1,000 μl DMSO solution of AAm (1,960 μmol), MBAAm (40 μmol) (total monomer concentration: 2.00 M) and AIBN (20 μmol). The weights of an AAm gel swollen in DMSO and H_2_O were 2,463 and 3,922 mg, respectively.

### Preparation of *t*Bu gels (*x*)

*t*Bu gels (*x*) were prepared according to a similar procedure for βCD–bpy gel, using 1,000 μl DMSO solution of AAm (1980−20*x *μmol), *t*BuAAm (20*x *μmol), MBAAm (20 μmol) (total monomer concentration: 2.00 M) and AIBN (20 μmol). The weights of a *t*Bu gel (10) swollen in DMSO and H_2_O were 5,125 and 6,155 mg, respectively.

### Observation of gels reacted with competitive hosts/guests

βCD–bpy gel was cut into *ca.* 1 × 1 × 1 mm pieces. Each piece was placed in a cylindrical well (φ 6.4 mm × 10 mm, polystyrene), 60 μl of H_2_O (depth ~2 mm) was added. Each piece of the gel was observed by the microscope and its size was measured. After that, 240 μl of a 12.5 mM aqueous solution of a competitive host (βCD) or a competitive guest (AdCANa: 1-adamantanecarboxylic acid sodium salt) was added to the wells (total 300 μl, 10.0 mM, 3 μmol, ~50 eq [/bpy or βCD]). Each piece of the gel was observed by the microscope 12 h after immersion and its size was measured. The experiments were performed at 25 °C.

### Observation of gels reacted with metal salts

βCD–bpy gel was cut into *ca.* 1 × 1 × 1 mm pieces. Each piece was placed in a cylindrical well (φ 6.4 × 10 mm, polystyrene), 80 μl of H_2_O (depth ~2.5 mm) was added. Each piece of the gel was observed by the microscope and its size was measured. After that, 16 μl of 10 mM aqueous solution of MCl_2_ (M=Mg, Mn, Fe, Co, Ni, Cu or Zn) was added to the wells (total 96 μl, 1.7 mM, 0.16 μmol, ~3 eq [/bpy]). Each piece of the gel was observed by the microscope 12 h after immersion, and its size was measured. The experiments were performed at 25 °C.

### Tensile modulus measurements of βCD–bpy gel and *t*Bu gel

A representative procedure: βCD–bpy gel was cut out into *ca.* 4 × 2 × 10 mm cuboids using a razor. Weight of each piece of the βCD–bpy gel was measured (*ca.* 80 mg, bpy 32 μmol). Each piece of the βCD–bpy gel and the *t*Bu gel was immersed in 10 mM CuCl_2_ aqueous solution (*ca.* 0.8 ml, amount of Cu^2+^ 2–3 eq [/bpy]) for 3 h. After immersion, the tensile strength measurement was performed using a creep metre (Yamaden, RE2-33005C. Load cell: 2 N (10-fold amplified sensitivity)). Sides of two pieces of the gel was clipped on the jig so that the tensile direction was vertical to its 4 × 2 mm face. The size of the sectional area was measured by a vernier caliper. Tensile stress-strain curves were measured as being pulled at a speed of 0.1 mm s^–1^. Young’s moduli of the gels were obtained from the initial gradients of the measurements.

### Qualitative adhesion tests of βCD–bpy gels and tBu gels

**Figure 4:**
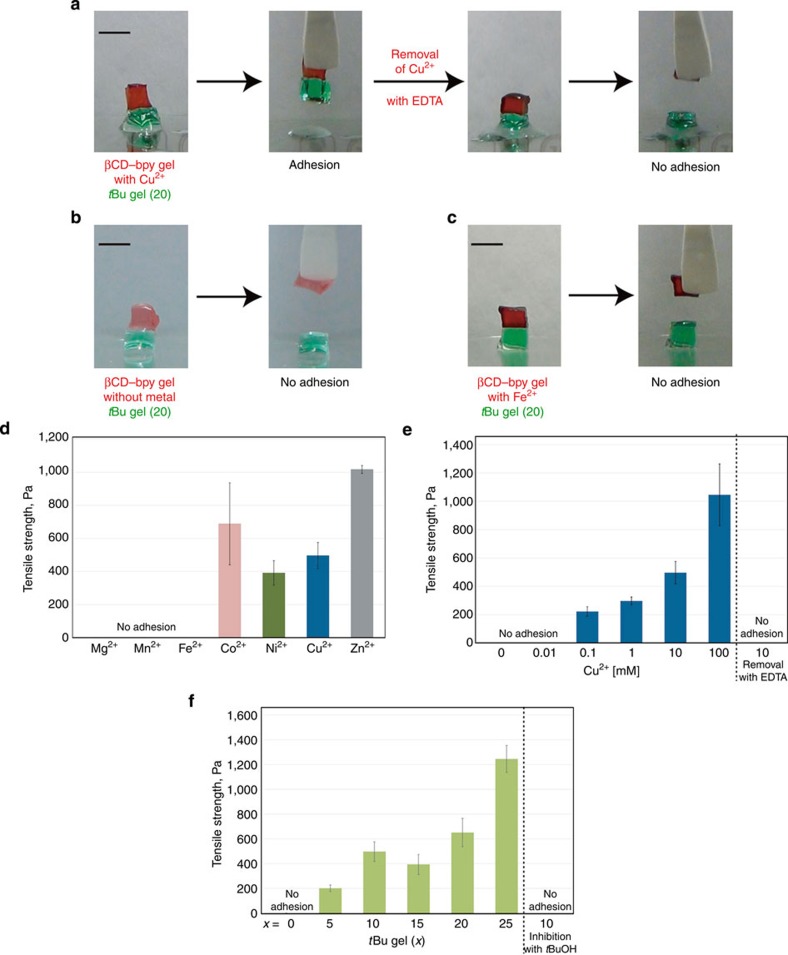
Metal–ion-responsive adhesion of βCD–bpy gel to *t*Bu gels. (**a**–**c**) Photographs of metal–ion-responsive adhesion between two pieces of βCD–bpy gel and *t*Bu gel (20). Each piece of gel was coloured with dyes for clarity (βCD–bpy gel; red. *t*Bu gel (20); green). Scale bars, 5 mm. (**a**) A piece of βCD–bpy gel immersed in CuCl_2_ aqueous solution adhered to that of *t*Bu gel (20). Removal of Cu^2+^ from βCD–bpy gel by immersion in an aqueous solution of EDTA·4Na switched off its adhesion ability. (**b**) A piece of βCD–bpy gel did not adhere to that of *t*Bu gel (20) without immersing in metal solutions. (**c**) A piece of βCD–bpy gel immersed in FeCl_2_ aqueous solution did not adhere to that of *t*Bu gel (20). (**d**–**f**) Tensile adhesive strength between two pieces of βCD–bpy gel and *t*Bu gel (*x*) immersed in metal salt solutions (error bars, s.e.m. (*n*=3)). (**d**) Immersion in various metal salt solutions (*t*Bu gel (10), [MCl_2_]=10 mM (M^2+^=metal ion, 3 eq [/bpy])). (**e**) Immersion in CuCl_2_ aqueous solutions with different concentrations (*t*Bu gel (10), [CuCl_2_]=*x* mM (Cu^2+^, 2*x* eq [/bpy])), and treatment of gels preimmersed in a 10 mM solution of CuCl_2_ with a solution of EDTA·4Na as a metal chelator. (**f**) *t*Bu gels (*x*) with different mol% contents of *t*Bu groups ([CuCl_2_]=10 mM (3 eq [/bpy])) and treatment of gels with an aqueous solution of *t*BuOH as an inhibitor of the host–guest complex.

A representative procedure: βCD–bpy gels and *t*Bu gels (20) were cut out into *ca.* 5 × 5 × 5 mm cubes using a razor. For clarity, pieces of βCD–bpy gel were dyed by immersing in *ca.* 40 ml of an aqueous solution of a red food colouring dye (containing 85 wt% of dextrins and 15 wt% of New Coccin, [New Coccin]=0.05 mM) and those of *t*Bu gel (20) were dyed by immersing in *ca.* 40 ml of an aqueous solution of a green food colouring dye (containing 88 wt% of dextrins, 8.4 wt% of Tartrazine and 3.6 wt% of Brilliant Blue FCF, (Tartrazine+Brilliant Blue FCF)=0.10 mM), respectively. Surfaces of both gels were rinsed with H_2_O before use. A piece of *t*Bu gel (20) was put on a glass petri dish. A piece of βCD–bpy gel was stacked on top of that of *t*Bu gel (20), and lifted up again after waiting for several seconds, showing that no adhesion between them. Next, the piece of βCD–bpy gel was put in 5 ml of a 100 mM aqueous solution of CuCl_2_ for 1 min. The piece of βCD–bpy gel immersed in CuCl_2_ solution was stacked on top of that of *t*Bu gel (20), and lifted up again after waiting for several seconds, showing that the adhesion was so strong that the piece of *t*Bu gel (20) was able to be picked up. After separating two gels using tweezers, the piece of βCD–bpy gel was immersed in 5 ml of a 100 mM aqueous solution of EDTA·4Na for 1 min. The piece of βCD–bpy gel did not show adhesive ability to that of *t*Bu gel (20) any more (see also Fig. 4a–c and Supplementary Movie 1).

### Tensile adhesion strength measurements

A representative procedure: βCD–bpy gel and *t*Bu gel (10) were cut out into *ca*. 5 × 2 × 10 mm cuboids using a razor. Weight of the βCD–bpy gel was measured (*ca*. 100 mg, bpy 40 μmol). Both βCD–bpy gel and *t*Bu gel (10) were immersed in a 10-mM aqueous solution of CuCl_2_ (*ca*. 1.2 ml, amount of Cu^2+^ 3 eq [/bpy]) for 3 h. After immersion, the tensile strength measurement was performed using a creep metre (Yamaden, RE2-33005C. Load cell: 2 N (10-fold amplified sensitivity)). Each side of two gels was clipped on the jig, and contacted on a 5 × 2 mm face. The size of the adhesion face was measured by a vernier caliper. Tensile adhesion strength at rupture was measured as being pulled at a speed of 0.1 mm s^–1^.

## Author contributions

T.N. and A. Harada conceived the project. T.N. designed and performed the experiments. T.N., Y.T. and A. Harada analysed the data and co-wrote the paper. A. Hashidzume and H.Y. contributed to discussion of the result.

## Additional information

**How to cite this article:** Nakamura, T. *et al*. A metal–ion-responsive adhesive material via switching of molecular recognition properties. *Nat. Commun.* 5:4622 doi: 10.1038/ncomms5622 (2014).

## Supplementary Material

Supplementary Information Supplementary Figures 1-9, Supplementary Tables 1-2 and Supplementary References

Supplementary Movie 1 Metal-ion-responsive adhesion between βCD-bpy gel and *t*Bu gel (20). A piece of βCD-bpy gel immersed in CuCl2 aqueous solution adhered to that of tBu gel (20). Removal of Cu^2+^ from βCD-bpy gel by immersion in an aqueous solution of EDTA·4Na switched off its adhesion ability.
