# Supplementary material for: A metal–ion-responsive adhesive material via switching of molecular recognition properties
Source: Nat Commun. 2014 Aug 7;5:4622. doi: 10.1038/ncomms5622 (PMC4143919; doi:10.1038/ncomms5622)
Supplement: Supplementary Information — Supplementary Figures 1-9, Supplementary Tables 1-2 and Supplementary References [file ncomms5622-s1.pdf]

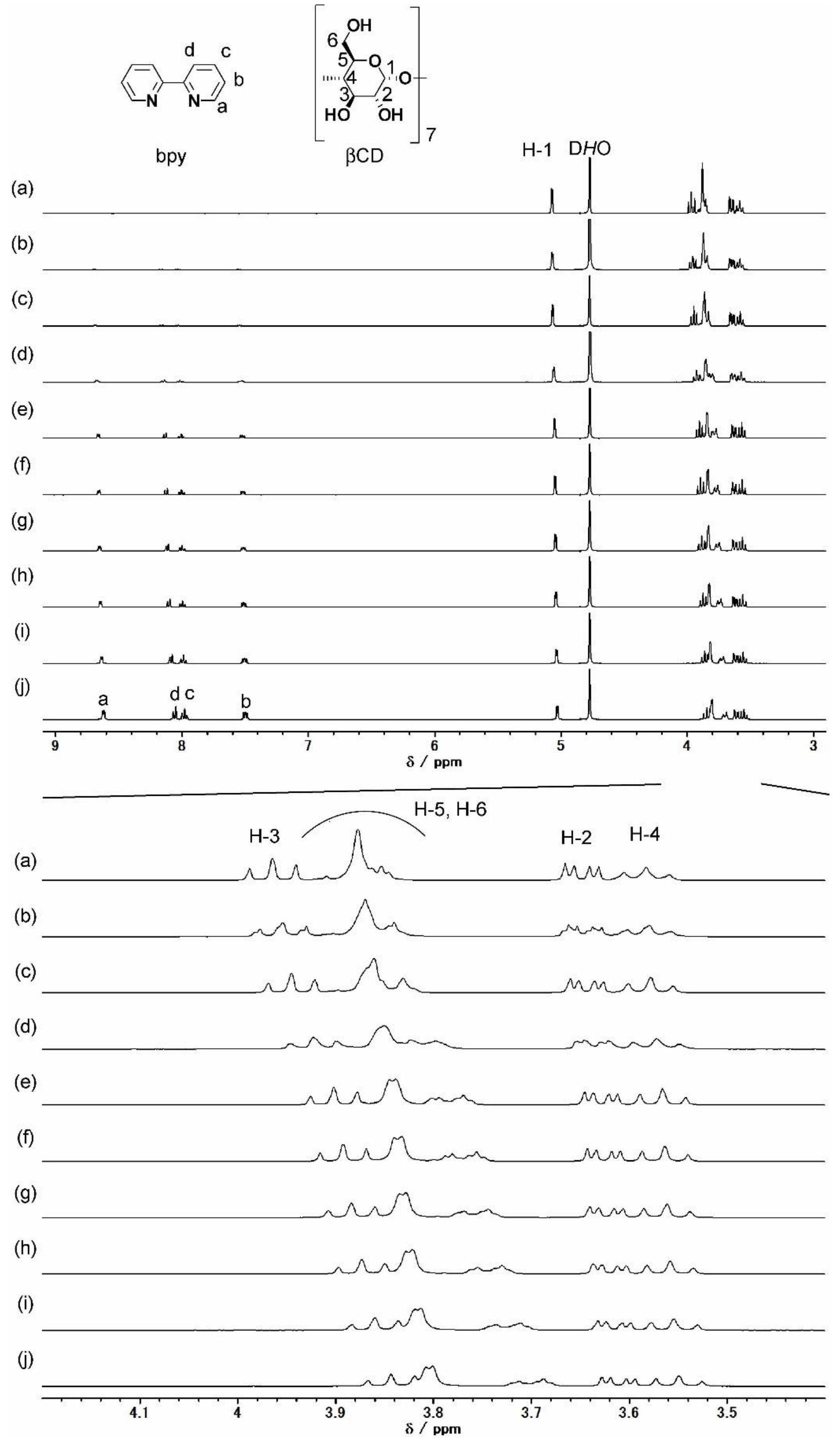

Supplementary Figure 1. A guest binding experiment of 2,2′-bipyridyl to βCD. Initial sample solution: [βCD] = 9.01 mM in D_2O. Stock solutions for titration: [bpy] = 50.0 mM in D_2O. Stock solution in D_2O was titrated into the sample solution. Each ^1H NMR measurement was performed within 5 min after each addition of stock solution. a)–j) ^1H NMR spectra. (400 MHz, 298 K, D_2O). a) βCD. b) (a) + 2,2′-bipyridyl, 0.1 eq. [/βCD]. c) 0.2 eq. d) 0.6 eq. e) 0.9 eq. f) 1.1 eq. g) 1.3 eq. h) 1.7 eq. i) 2.2 eq. j) 3.3 eq.

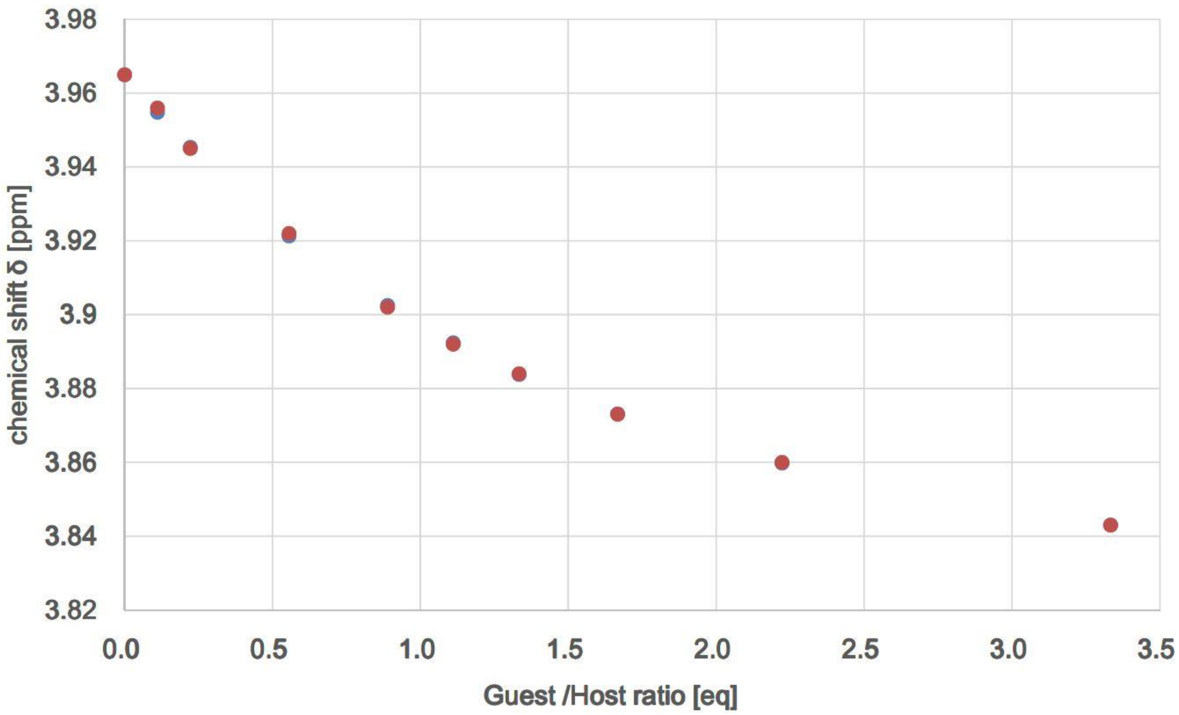

Supplementary Figure 2. The chemical shift changes of ^{1}H NMR signal of H-3 in βCD in the titration experiment of 2,2′-bipyridyl against βCD (Supplementary Figure 1). Red squares denote the experimental values. Blue circles denote the calculated values with ($K_a = 1.0 \times 10^2$ M^{-1}, δ(βCD⊃bpy) = 3.764 ppm).

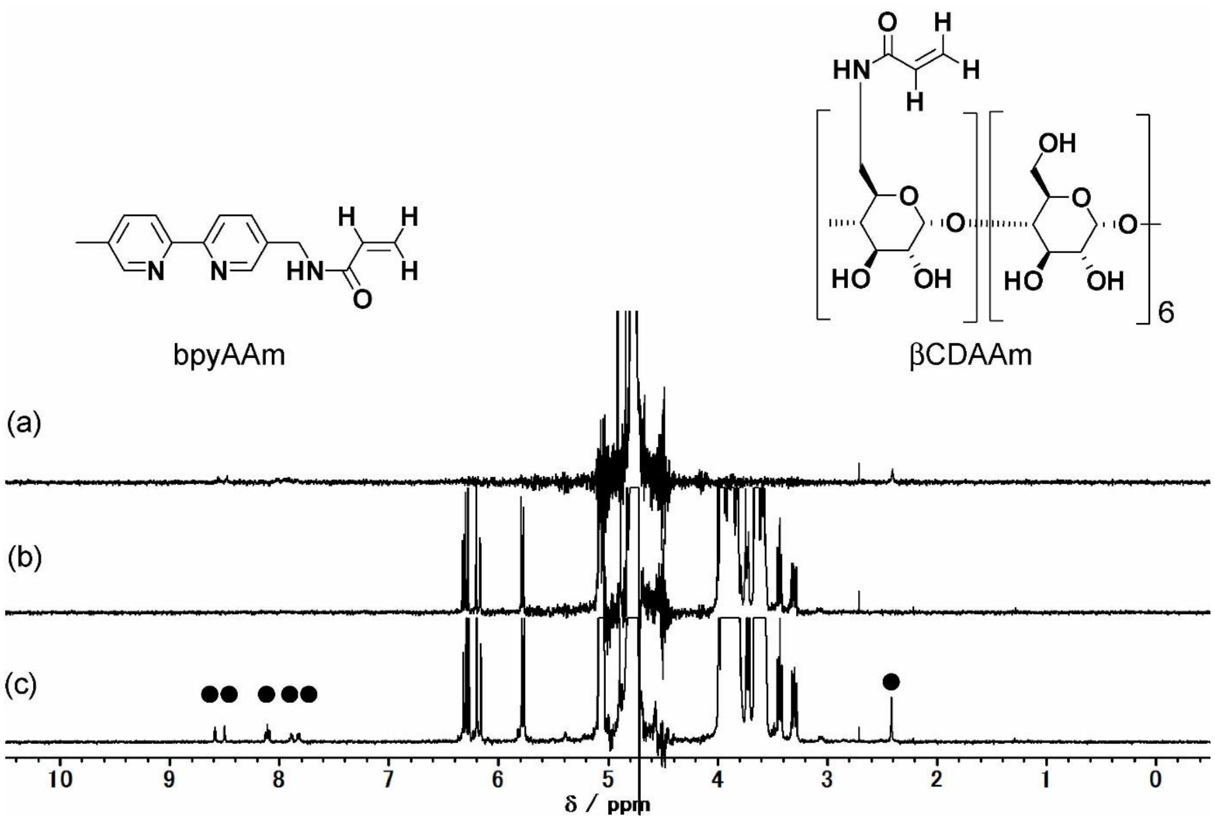

Supplementary Figure 3. An inclusion experiment of bpyAAm into βCDAAm. a)–c) ^{1}H NMR spectra (500 MHz, D_2O, 298 K). a) bpyAAm suspended in D_2O. b) [βCDAAm] = 10.5 mM in D_2O. c) (a) + βCDAAm, 1.0 eq. [/βCDAAm], [βCDAAm] = 10.5 mM. Filled circles indicate the signals of bpyAAm encapsulated in βCDAAm. Formation ratio of bpyAAm⊂βCDAAm was 10% in this condition.

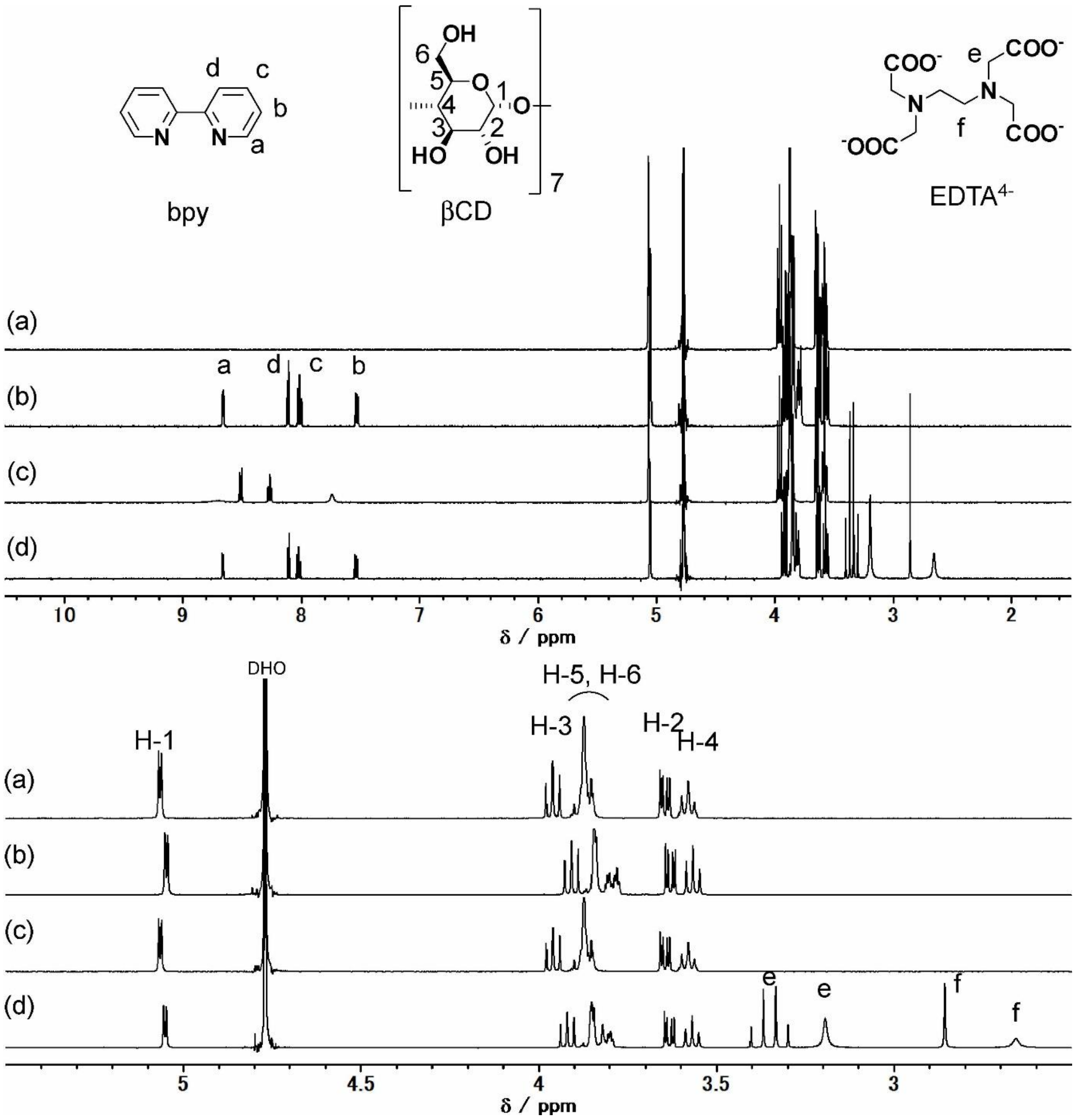

Supplementary Figure 4. Control of encapsulation of βCD⊂bpy by Zn^{2+} ion. . a)–d) ^{1}H NMR spectra. (500 MHz, 298 K, D_2O). Magnification of vertical axis of upper and lower figures were changed for clarity. (a) Initial sample solution: D_2O solution of βCD (5.0 mM, 500 μL, 2.5 μmol, 1.0 eq.). (b) D_2O solution of 2,2′-bipyridyl (bpy, 50.0 mM, 50 μL, 2.5 μmol, 1.0 eq.) was added to form an inclusion complex, bpy⊂βCD. (c) D_2O solution of $ZnCl_2$ (30.0 mM, 125 μL, 3.75 μmol, 1.5 eq.) was added to form Zn-bpy complexes and dissociate the inclusion complex βCD⊃bpy. (d) D_2O solution of EDTA·4Na (100 mM, 75 μL, 7.5 μmol, 3.0 eq.) was added to remove Zn^{2+} and regenerate the inclusion complex βCD⊃bpy.

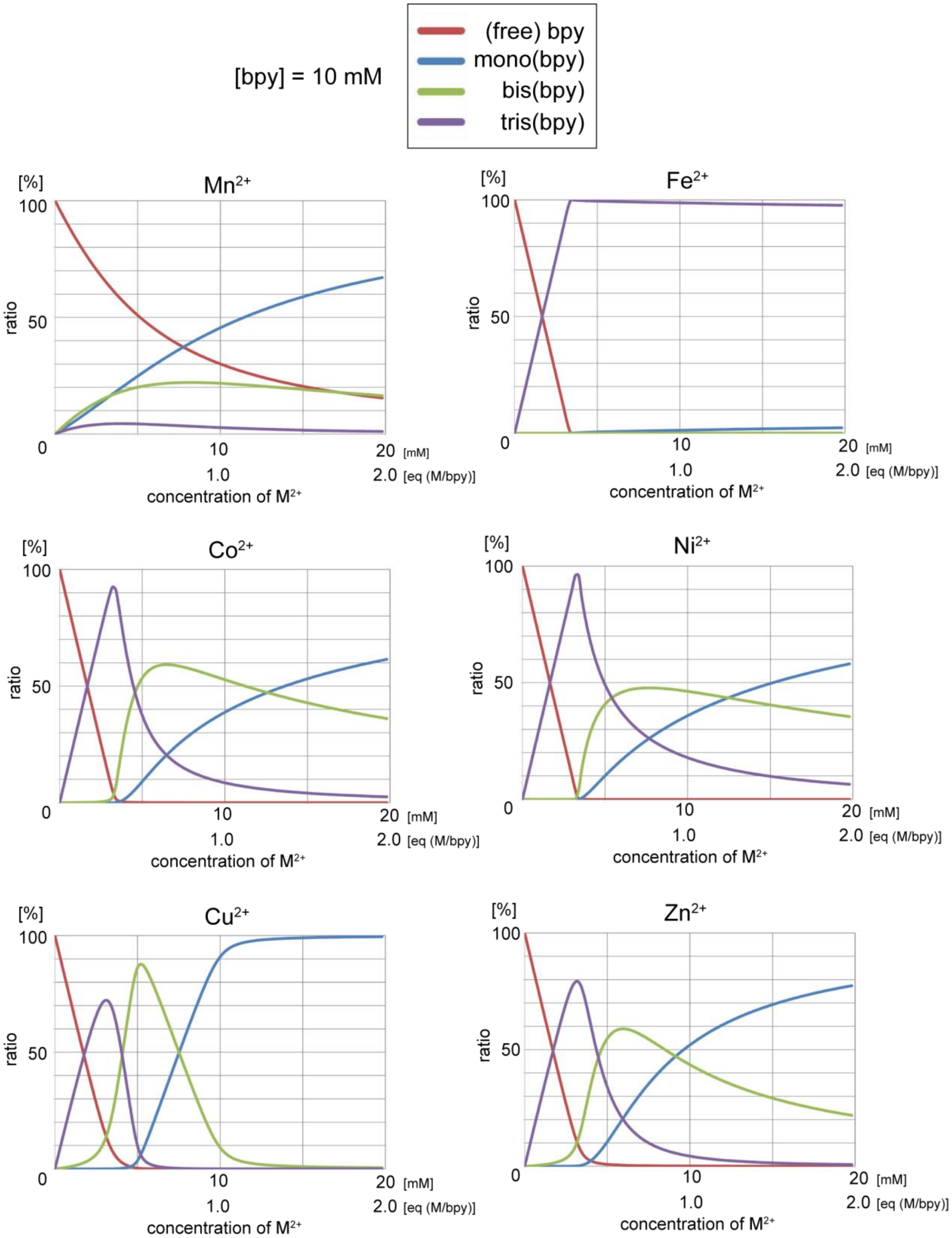

Supplementary Figure 5. Visualization of the relationship between concentrations of metal ions and equilibrium of metal-bpy complexes (In the case of [bpy] = 10 mM. Using the equilibrium constants of Supplementary Table 2).

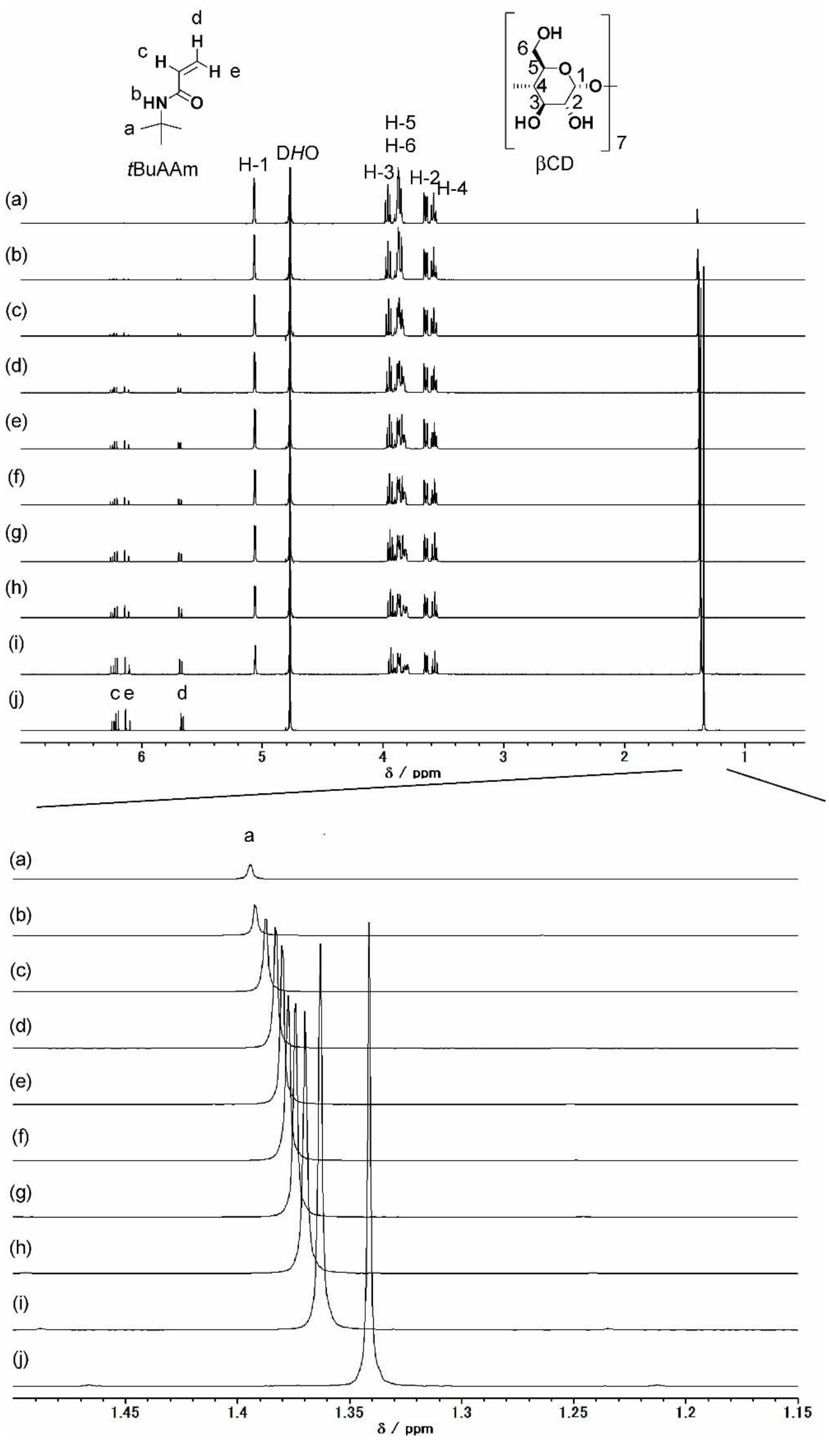

Supplementary Figure 6. A guest binding experiment of *t*BuAAm to βCD. Initial sample solution: [βCD] = 10.0 mM in D_2O. Stock solutions for titration: [*t*BuAAm] = 50.0 mM in D_2O. Stock solution in D_2O was titrated into the sample solution. Each ^{1}H NMR measurement was performed within 5 min after each addition of stock solution. a)–j) ^{1}H NMR spectra. (500 MHz, 298 K, D_2O). a) βCD + *t*BuAAm, 0.1 eq. [/βCD]. b) 0.2 eq. c) 0.5 eq. d) 0.8 eq. e) 1.0 eq. f) 1.2 eq. g) 1.5 eq. h) 2.0 eq. i) 3.0 eq. j) *t*BuAAm.

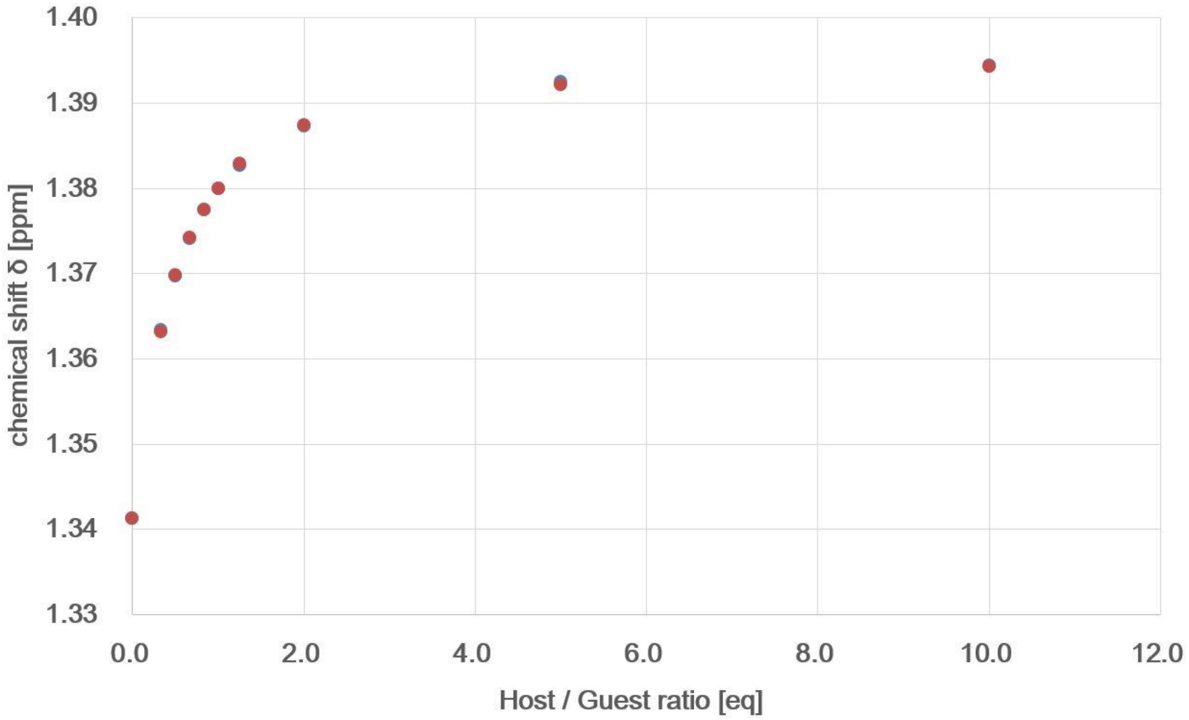

Supplementary Figure 7. The chemical shift changes of ^{1}H NMR signal of *t*Bu group of *t*BuAAm in the titration experiment of *t*BuAAm against βCD (Supplementary Figure 6). Red circles denote the experimental values. Blue circles denote the calculated values with ($K_a = 1.0 \times 10^2$ M^{-1}, δ(*t*Bu⊂βCD) = 1.453 ppm).

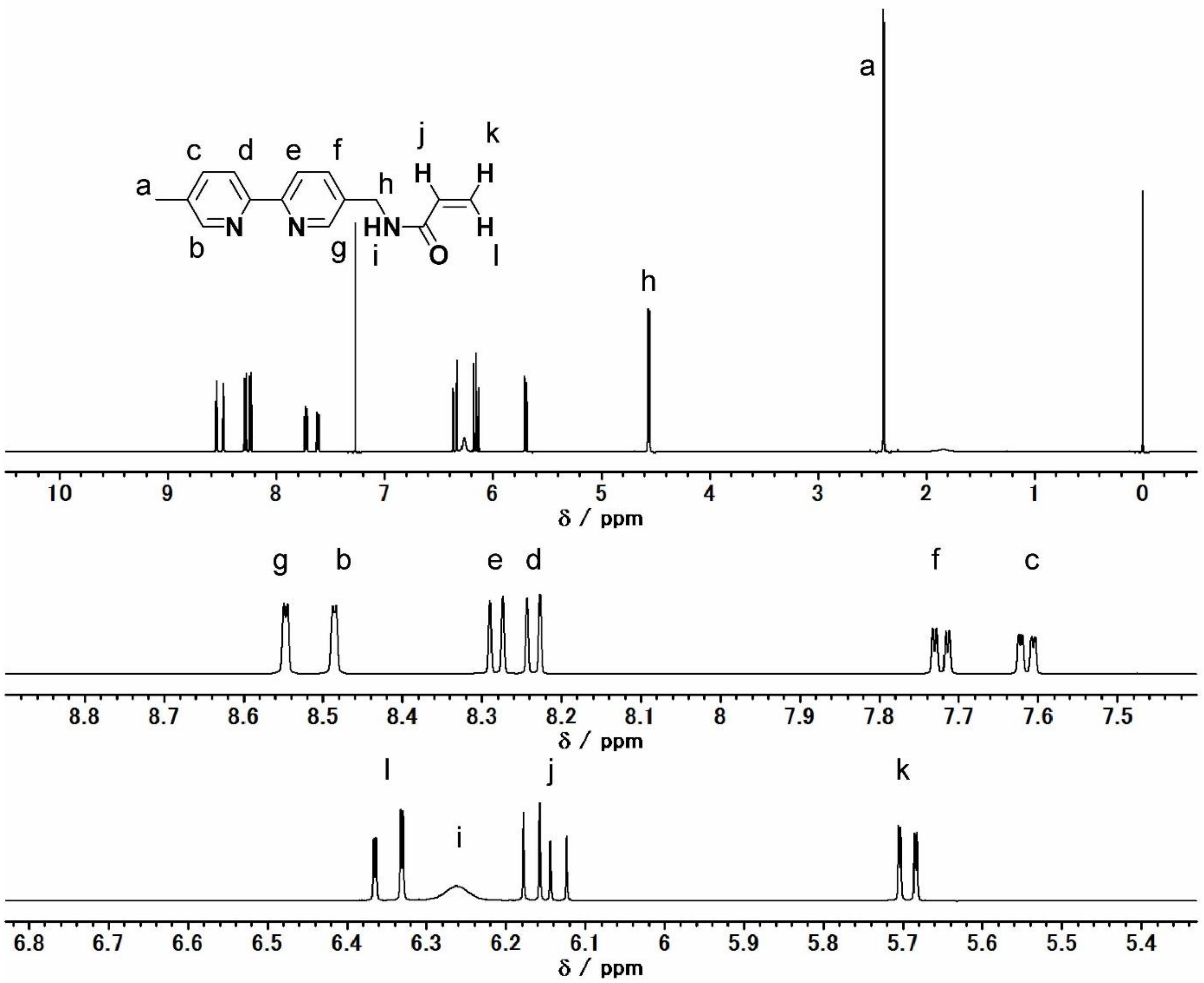

Supplementary Figure 8. ^{1}H NMR spectrum of bpyAAm (500MHz, 298 K, $CDCl_3$).

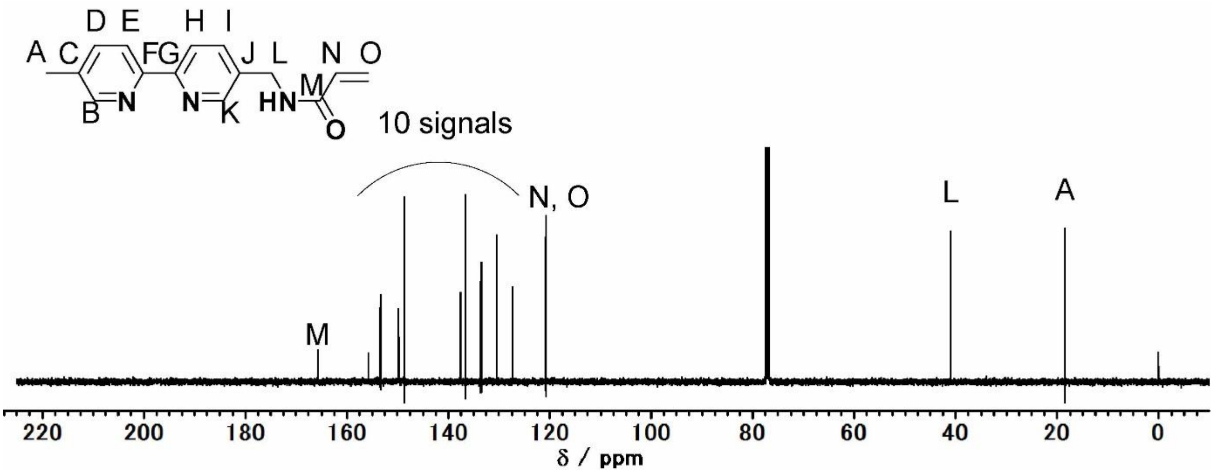

Supplementary Figure 9. ^{13}C NMR spectrum of bpyAAm (126MHz, 298 K, $CDCl_3$).

Supplementary Table 1: Weight and appearance of βCD-bpy gels (*x*,*y*,*z*). Each βCD-bpy gel (*x*,*y*,*z*) was prepared according to the procedure described on the Method section in the main text, with 300 μL of DMSO solution (total monomer concentration: 2.00 M), and the feed ratio of each monomer as follows: AAm: (100–*x*–*y*–*z*) mol%, βCDAAm: *x* mol%, bpyAAm: *y* mol%, MBAAm: *z* mol%. Gels were washed and swollen enough with DMSO, and successively with H_2O, prior to each weight measurement. βCD-bpy gel (3,3,2) was clear, homogeneous, and had adequate hardness as hydrogels. Increasing the molar ratio of βCD and bpy lead to further contraction, which can be ascribed to the increase of supramolecular cross-linking of βCD⊃ bpy. Decreasing the MBAAm units also lead to inhomogeneous shrinking during the solvent replacement.

gel	DMSO gel [mg]	H_2O gel [mg]	wt% change H_2O/DMSO	appearance of H_2O gel
βCD-bpy gel (3,3,1)	1583	286	18%	A
βCD-bpy gel (4,4,1)	1897	211	11%	B
βCD-bpy gel (5,5,1)	2352	178	8%	C
βCD-bpy gel (2,2,2)	941	392	42%	A
βCD-bpy gel (3,3,2)	1046	331	32%	A
βCD-bpy gel (4,4,2)	1338	240	18%	A
βCD-bpy gel (5,5,2)	1577	205	13%	B
βCD-bpy gel (7,7,2)	1835	162	9%	C
βCD-bpy gel (5,5,4)	1577	205	13%	A

Appearance of H_2O gels:

A ··· Colorless, clear at room temperature.

B ··· UCST-type phase separation around 20 °C (The gel was turbid at temperatures < 20 °C and clear at temperatures > 20 °C).

C ··· Inhomogeneously contracted after replacement of solvent to H_2O.

Supplementary Table 2: Complexation constants of 2,2′-bipyridyl and transition metal ions taken from the literature. (H_2O, 25 °C, 0.1 M Cl^-, NO_3^-, or ClO_4^-) Values of Fe^{2+} were taken from Supplementary Reference 1. The other values were from Supplementary Reference 2.

Metal ions	log K_1	log K_2	log K_3	log β_3
Mn^{2+}	2.55	1.9	1.45	5.9
Fe^{2+}	4.2	3.7	9.55	17.45
Co^{2+}	7.0	5.5	4.7	16.0
Ni^{2+}	8.2	6.8	6.4	20.2
Cu^{2+}	5.2	5.6	3.4	17.2
Zn^{2+}	4.3	4.5	3.7	13.4

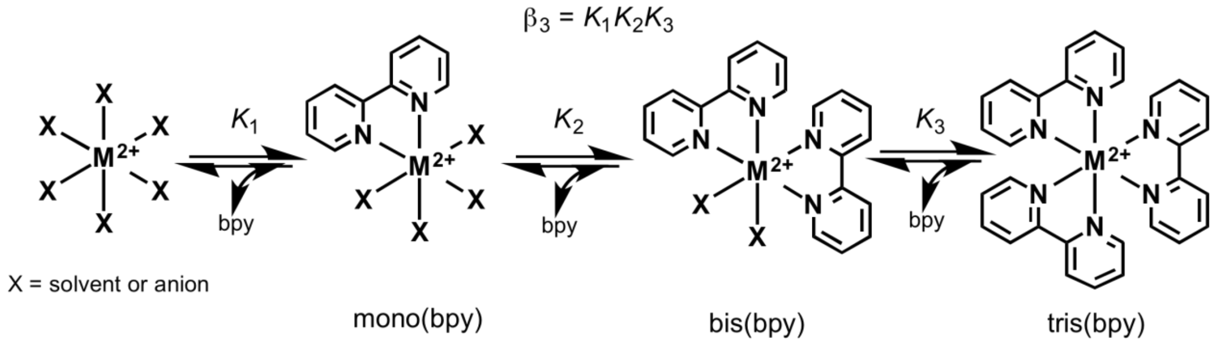

Supplementary References

1. Irving, H. & Mellor, D. H. The stability of metal complexes of 1,10-phenanthroline and its analogues. part I. 1,10-phenanthroline and 2,2´-bipyridyl. *J. Chem. Soc.* 5222-5237 (1962).

2. McBryde, W.A. E. A Critical Review of Equilibrium Data for Proton- and Metal Complexes of 1,10-Phenanthroline, 2,2′-Bipyridyl and Related Compounds (Pergamon Press Ltd., Oxford, 1978).
